# Invasive *Streptococcus intermedius* Infections in Children: Two Cases from a Pediatric Infectious Diseases Unit in Italy

**DOI:** 10.3390/pathogens13121099

**Published:** 2024-12-12

**Authors:** Piero Veronese, Simone Cella, Alessandra Giacometti, Irene Lapetina, Valentina Maffini, Marco Pappalardo, Monica Rubini, Maria Beatrice Ruozi, Icilio Dodi

**Affiliations:** 1 Pediatric Infectious Disease Unit, Children’s Hospital of Parma, 43126 Parma, Italy; agiacometti@ao.pr.it (A.G.); ilapetina@ao.pr.it (I.L.); vamaffini@ao.pr.it (V.M.); mpappalardo@ao.pr.it (M.P.); rubinim@ao.pr.it (M.R.); mruozi@ao.pr.it (M.B.R.); idodi@ao.pr.it (I.D.); 2 Pediatric Radiology Unit, Institute of Radiology, University of Parma, 43126 Parma, Italy; scella@ao.pr.it

**Keywords:** *Streptococcus* intermedius, acute mediastinitis, liver abscess, pediatric invasive infections

## Abstract

In recent years, an increasing number of reports have described invasive infections caused by bacteria from *Streptococcus anginosus group* (SAGs). *S. intermedius* seems to be more related with pleuropulmonary infections and abscess of the brain and deep soft tissues, and it is more likely to cause suppurative and non-bacteremic infections compared to other members of the same genus. We present two clinical cases of invasive *S. intermedius* infections in pediatric patients: a liver abscess case and a pansinusitis case associated with bilateral otomastoiditis and parapharyngeal abscess complicated by acute mediastinitis, thrombophlebitis of the cavernous sinus, and thrombosis of the cranial tract of the ipsilateral jugular vein. In both cases, prompt broad-spectrum antibiotic therapy and operative drainage of the collections resulted in a good clinical response with full recovery.

## 1. Introduction

*Streptococcus intermedius* is a facultatively anaerobic, Gram-positive, non-motile, catalase-negative member of the *Streptococcus anginosus group* (SAG) with two other species: *Streptococcus anginosus* and *Streptococcus constellatus* [1]. This group was previously named “*milleri*” in honor of W.D. Miller, but the term SAG proposed by Kawamura is currently the most widely accepted [1].

SAGs are often isolated from oral structure samples; indeed, *S. intermedius* is frequently associated with periodontal disease [2,3]. SAG members are commonly isolated from the throat, nasopharynx, and respiratory and gastrointestinal tracts [4]. *S. intermedius*, like other SAG members, has been considered only part of commensal microbiota for many years and not a relevant source for infections [5]. In recent years, an increasing number of reports have described invasive infections by SAGs, probably due to ongoing improvements in the sensitivity of diagnostic tests [6]. At the moment, SAGs could be classified as opportunistic pathogens, especially in immunocompromised patients or those with cancer or cystic fibrosis [7,8]. Specifically, *S. intermedius* is the causative agent of pleuropulmonary infections, including abscess of the brain and deep soft tissues [9,10,11].

Streptococcal virulence comprises multiple steps, including adhesion to the host tissue, invasion, and colonization. Adhesion is mediated by fibronectin-binding proteins (FBPs) and laminin-binding proteins (LBPs) [12,13]; capsule polysaccharide (CPS) and hemolysins are key virulence factors mediating invasion and colonization [14,15,16]. Intermedilysin (ILY) and sialidase A (NanA) are *S. intermedius*-specific virulence factors [10,17].

Invasive infections, including abscesses and empyema formation, can result from the dissemination of SAGs to sterile tissues [7]. *S. intermedius* is likely to cause suppurative infections; other SAGs are frequently associated with bacteremia rather than suppurative infections [18].

Malignancies (both solid and hematological), type 2 diabetes mellitus, chronic organ failure (heart, lung, and kidney), and chronic liver diseases (including chronic viral hepatitis) are established risk factors for the development of invasive infections. Mucosal lesions (inflammation and ulceration of gingival and periodontal tissues, peptic ulcer disease, surgical interventions, inflammatory bowel diseases, and intravenous drug use) can facilitate the systemic spread of germs into the bloodstream [19].

SAG-related invasive infections include brain, peri-tonsillar, and orofacial abscesses; Lamierre’s syndrome (septic thrombophlebitis of the internal jugular vein); pneumonia with empyema and lung abscesses; pericarditis; endocarditis; and liver abscesses [20,21,22,23,24,25,26,27].

In a pediatric population, Ismail et al. (2022) showed that *S. intermedius* was frequently associated with head and neck infections, probably due to the higher rate of upper respiratory tract infections [28].

*S. intermedius* is usually susceptible to beta-lactams [29].

We report two clinical cases of *S. intermedius* invasive infections in children from our recent experience in a Pediatric Infectious Disease unit: a liver abscess in a 7-year-old boy and a disseminated infection with pansinusitis, bilateral mastoiditis, parapharyngeal abscess, and acute mediastinitis associated with thrombophlebitis of the left cavernous sinus and jugular vein thrombosis in a 14-year-old girl.

Liver abscesses are usually polymicrobial infections. Meddings et al. (2010) highlighted *Streptococcus* as one of the most frequently isolated species from culture samples [30].

In addition, Gram-negative (*Escherichia coli*, *Klebsiella pneumoniae*, *Pseudomonas* spp., *Proteus* spp., *Salmonella*, *Shigella*) and anaerobic bacteria (*Bacterioides* spp., *Fusobacterium*) can cause hepatic and biliary tract infections. *Actinomyces*, *Candida* spp., and protozoa (*Entamoeba hystolitica*) can also be involved [31].

Typically, bacterial sinusitis is a mild disease. *S. intermedius* has been reported to cause more severe intracranial sinusitis-related complications than other bacterial species [32,33]; complications include Pott’s puffy tumor, orbital cellulitis, subdural/epidural empyema, meningitis, and venous sinus thrombosis [34].

Parapharyngeal abscess occurs due to an infection spreading along the fascial planes of the neck from a contiguous local site [35]. Parapharyngeal space abscess can rapidly lead to other conditions, such as Horner syndrome, and life-threatening complications, such as Lamierre’s syndrome and acute mediastinitis [36].

## 2. Case Reports

### 2.1. Case One—Liver Abscess

#### 2.1.1. Medical History and Clinical Manifestations

A 7-year-old boy presented to the Emergency Department of the Children Hospital of Parma, Italy, with a 4-day history of high-grade fever, diarrhea, vomiting, and abdominal pain.

Antibiotic therapy with amoxicillin plus clavulanate acid was previously prescribed by a general practitioner (a total of four doses had been taken).

The past medical history of the boy was unremarkable. Growth and development had always been regular. He has no history of previous invasive or recurrent infections. His travel history and food exposure history were unremarkable.

#### 2.1.2. Initial Assessment and Hematological and Radiological Investigations

Upon arrival, the patient was without fever and in a good general condition but reported acute abdominal pain. Vital signs were normal. The abdomen was soft with tenderness in the upper right part. A general examination revealed no notable findings. A SARS-CoV2 nasopharyngeal swab was negative. Blood exams were performed and showed leukocytosis with a high presence of neutrophiles and high levels of CRP and procalcitonin, as described in Table 1. Initial investigations also revealed a mild elevation in aspartate and alanine aminotransferase (AST 81 U/L and ALT 120 U/L; normal range is 0.0–45 U/L) with tests showing normal liver function and no evidence of cholestasis. An ultrasound of the abdomen showed a large 70 × 50 cm predominantly hypoechoic and fluid-containing liver lesion with vascularization in the right hepatic lobe. The imaging features were highly suggestive of hepatic abscess.

**Figure 1:**
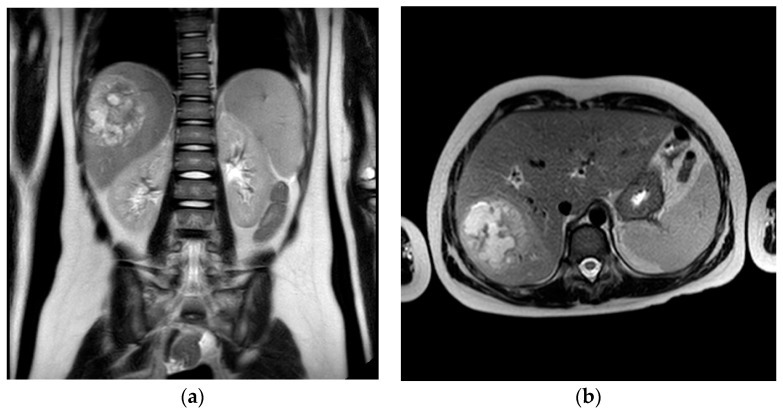
T2-weighted coronal (**a**) and axial (**b**) MRI images acquired before percutaneous drainage. of the abscess in the upper right hepatic lobe. Presence of the large hepatic abscess in the upper section of the right hepatic lobe, appearing as a hyperintense lesions.

Subsequent MRI of the abdomen confirmed the T2-hyperintense and T1-hypointense lesion in the VII hepatic segment with dimensions of 65 × 53 × 74 mm (AP × LL × CC) (Figure 1).

Upon admission to our ward, broad-spectrum antibiotic therapy was started with ceftriaxone and metronidazole.

#### 2.1.3. Microbiology Investigations

Blood, stools, and urinary samples were collected for a microbiological examination (FilmArray-PCR and cultures) without positive results. An interferon gamma release assay (Quantiferon TB Gold Plus) excluded tuberculosis infection.

A drainage sample of the hepatic lesion revealed some Gram-positive cocci in direct microscopy; the 72 h culture confirmed the presence of Gram-positive bacteria. Species identification by MALDI-TOF mass spectrometry confirmed the isolate as *Streptococcus intermedius*. Susceptibility testing was performed: the isolate was only resistant to tetracycline, while it was susceptible to ampicillin, penicillin, ceftriaxone, clindamycin, erythromycin, levofloxacin, linezolid, vancomycin, and chloramphenicol. Unfortunately, the minimum inhibitory concentration (MIC) and minimum bactericidal concentration (MBC) are not reported in the results.

#### 2.1.4. Treatment and Outcome

On day 2, the patient underwent ultrasound-guided needle drain of the abscess. No percutaneous drainage was placed. The drainage fluid did not have positive results for cytological atypia, and the culture isolated *S. intermedius*. Additional drainage was performed on day 8: a microbiological examination of the aspiration fluid was repeated but resulted in a negative result for *S. intemedius*; at the same time, a specimen for biopsy was collected, and histopathological assays showed phlogistic and necrotic tissue. Fever disappeared after 11 days of antibiotic therapy; after that, during the remainder of the hospitalization period, no relapses of fever were observed.

In parallel, a blood exam revealed a gradual improvement in CPR and PCT levels. PCT became negative on day 8 of admission, and CRP on day 16. Hepatic transaminases normalized immediately after admission. No significant laboratory signs of cholestasis were observed. An ultrasound follow-up was performed, showing no relapses of the lesion. The US exam performed at discharge highlighted a maximum diameter of 40 mm.

The patient was discharged with an additional 3 weeks of wide-spectrum antibiotic therapy (amoxicillin + clavulanate acid); the total duration of antibiotic therapy was 5 weeks.

**Figure 2:**
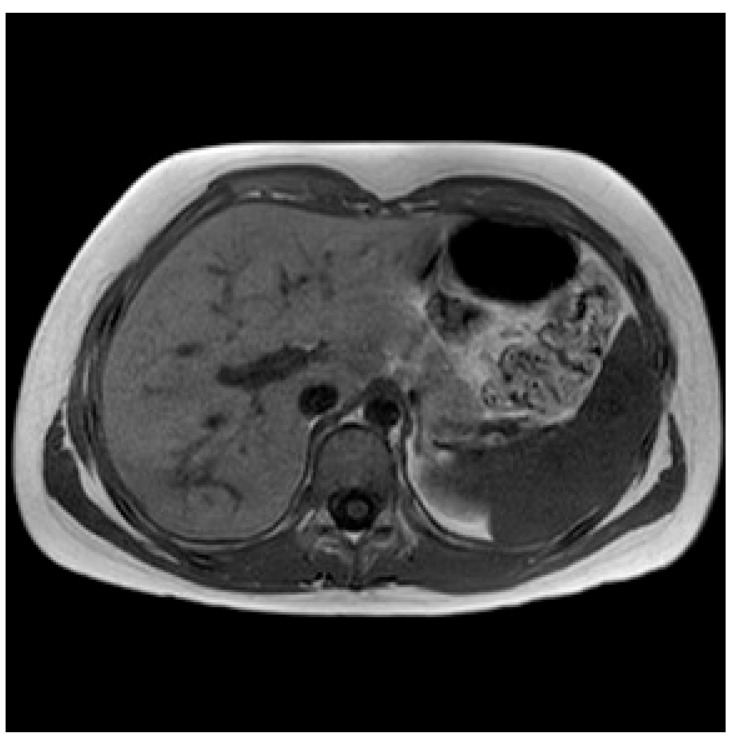
A T1-weighted follow-up MRI image after 6 months, revealing the complete resolution of the previous hepatic lesion.

MRI was repeated 5 months after discharge and showed the complete resolution of the abscess (Figure 2).

One year post discharge, the patient remains free from evidence of relapse of infection.

### 2.2. Case Two—Pansinusitis, Bilateral Otomastoiditis, and Parapharyngeal Abscess Complicated by Acute Mediastinitis, Thrombophlebitis, and Vein Thrombosis

#### 2.2.1. Medical History and Clinical Manifestations

A 14-year-old girl presented to the emergency department of another district in Northern Italy with a 4-day history of throbbing headache associated with photophobia. After a neurological evaluation, the patient was discharged on a regimen of as-needed analgesics. After 3 days, the girl returned to the same emergency department due to the persistence of symptoms associated with fever and bilateral otalgia. She presented with fever (temperature 39 °C), moaning, severe right-sided headache, severe photophobia and phonophobia, and neck stiffness.

The past medical history of the girl was silent. Growth and development had always been regular. The patient denied any previous history of headaches and severe or recurrent infections.

#### 2.2.2. Initial Assessment and Hematological and Radiological Investigations

Due to the worsening of the clinical status, hematological and radiological investigations were promptly performed. Blood exams at admission showed marked leukocytosis (WBC of 30.97 × 10^9^/L and 27.23 × 10^9^/L neutrophils; WBC normal range of 4.5–11 × 10^9^/L) with elevated procalcitonin (PCT 2.45 ng/mL; normal value < 0.5 ng/mL) and increased pro-thrombotic markers (D-dimer 1833 ng/mL, normal value: <500 ng/mL; fibrinogen 890 mg/dL, normal range: 150–400 mg/dL). An urgent brain and neck CT scan was performed, which showed a right frontal-maxillary sinusitis with involvement of the ipsilateral ethmoid and sphenoid bones. Lower scans revealed a retro-pharyngeal abscess on the right side with the contemporary involvement of the adipose tissue of the paraspinal regions. A diagnostic lumbar puncture was then performed: cerebrospinal fluid (CSF) was clear with a normal pressure, and its examination highlighted pleocytosis (52 mg/dL proteins and 131/microL leukocytes; normal value of lymphocyte count of <5/microL, normal protein concentration of 20–45 mg/dL, and normal glucose concentration 50–100 mg/dL). The patient was then transferred to the Intensive Care Unit (ICU) of Parma.

**Figure 3:**
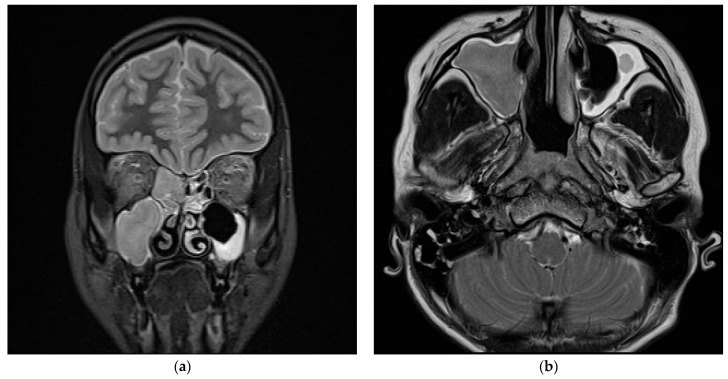
MRI T1-weighted hypointense image of sinusitis of the ethmoid and maxillary sinuses in the coronal plan (**a**). Concomitant hypointense signal in the mastoid regions is consistent with bilateral otomastoiditis (**b**).

**Figure 4:**
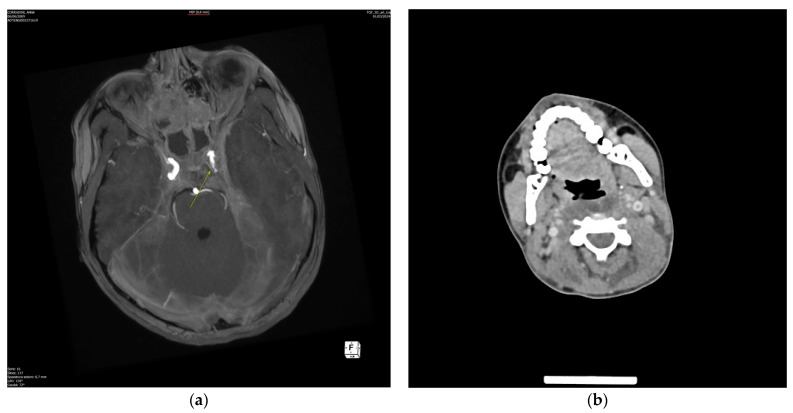
Axial images revealing the presence of thrombophlebitis of the left cavernous sinus (**b**) with partial thrombosis of the cranial tract of the ipsilateral jugular vein (**a**), appearing as contrast-enhanced filling defects.

At the ICU, magnetic resonance imaging (MRI) and Angio-MRI of the brain and the neck were performed, confirming pansinusitis and highlighting bilateral involvement of the mastoids; the angiographic images revealed the presence of thrombophlebitis of the left cavernous sinus with a partial thrombosis of the cranial tract of the ipsilateral jugular vein (Figure 3 and Figure 4). At the same time, a CT scan of the thorax and the neck confirmed a large left parapharyngeal abscess with an extension to the anterior part of the mediastinum.

#### 2.2.3. Microbiology Investigations

Microbiological examinations (direct microscopy, Film Array-PCR, and cultures) of CSF had negative results for virus, bacteria, and fungi. Blood samples were collected for microbiological investigations, and the 72 h culture isolated *Streptococcus intermedius*. A drug susceptibility test was performed in the laboratory of the first hospital and showed good susceptibility to penicillin; unfortunately, a copy of the result was not sent to us, so it is currently not available.

The purulent sample previously drained from the maxillary sinus isolated *Cutinebacterium acnes* (MALDI-TOF mass spectrometry), a saprophytic microorganism of the skin.

#### 2.2.4. Treatment and Outcome

Wide-spectrum antimicrobial therapy with intravenous ceftriaxone, ampicillin and acyclovir was promptly started after CT evidence of pansinusitis and parapharyngeal abscess of the neck. Upon admission to the ICU, ampicillin was switched with vancomycin; acyclovir was suspended as soon as a negative result of a virus in the CSF was confirmed.

On day 2, the patient underwent surgical drainage of the parapharyngeal abscess. No post-surgical complications were observed.

After 3 days in the ICU, the girl was transferred to our Pediatric Infectious Disease Unit; antimicrobial therapy with ceftriaxone and vancomycin was continued, and metronidazole was added.

Given the Angio-MRI evidence of thrombophlebitis of the cavernous sinus with partial thrombosis of the jugular vein, a therapeutic regimen with acetylsalicylic acid and enoxaparin was initiated following consultation with neurologists and radiologists in the stroke unit.

The patient had an uncomplicated postoperative course with gradual but progressive improvements in her clinical conditions; she was discharged after an 18-day stay in the ward.

After discharge, the patient continued periodic check-up visits at our clinic. Antibiotic therapy was switched to oral cefuroxime and metronidazole for a total duration of 42 days.

ASA and enoxaparin were stopped after MRI evidence of the resolution of known thrombosis and thrombophlebitis after 2 months of therapy. After a 6-month multi-specialist pediatric, infectious disease, neurological, radiological, and physical follow-up, the girl returned to her normal life without any neurological or functional deficits.

## 3. Discussion

Over the past few years, there has been an increase in reports of invasive bacterial infections in children, and great attention has been paid to recent alerts regarding the issue of *Streptococcus pyogenes* invasive infections [37,38,39,40]. Additionally, in recent decades, an increase in the number of invasive and/or disseminated infections by the *Streptococcus anginosus group* (SGA) has been described [8]. The increasing number of reports is shifting the perception of this group of bacteria, which are no longer considered mere commensals but potential pathogens.

We present two clinical cases of invasive *Streptococcus intermedius* infections observed in our Pediatric Infectious Disease Unit: a liver abscess in a 7-year-old boy and a case of pansinusitis associated with bilateral otomastoiditis and parapharyngeal abscess complicated by acute mediastinitis, thrombophlebitis of the cavernous sinus, and thrombosis of the cranial tract of the ipsilateral jugular vein.

Neither of our young patients had any local (oral lesions, infections, or procedures) or systemic risk factors, and neither had significant medical history [8]. As widely described, *S. intermedius* infections are primarily associated with the development of abscesses in soft tissues, the respiratory tract, and brain [41].

Issa et al. highlighted that the majority of invasive SAG infections originate from the oral mucosa and the upper respiratory tract, spreading through direct contiguity or hematogenous dissemination [10]. In case number one, it is highly probable that hepatic involvement is secondary to hematogenous spread of the microorganisms; it is less likely that it is secondary to the intestinal translocation of the bacteria. According to Livingston et al., most Streptococcal liver abscesses are preceded by dental procedures or infections of the oral cavity (e.g., periodontitis) and subsequent bacteriemia [42]. Nevertheless, hematogenous spread can occur even in the absence of active oral infections, as in our clinical case.

Regarding case number two, contiguous spread likely caused pansinusitis and bilateral otomastoidits, which were subsequently complicated with extension to deeper tissues.

Both cases presented with typical features of suppurative infections; clinically, malaise, fever with chills, and organ-related symptoms (abdominal pain in case one and headache in case two). The laboratory findings revealed a significant elevation in inflammatory markers and neutrophilic leukocytosis (case one: WBC of 15.28 × 10^9^/L, 81.7% neutrophils, CRP of 236.9 mg/L, and PCT concentration of 10.9 ng/mL; case two: WBC of 30.97 × 10^9^/L, 27.23 × 10^9^/L neutrophils, and PCT concentration of 2.45 ng/mL).

Targeted imaging studies confirmed the presence of deep-seated infections, allowing for the accurate identification, localization, and measurements of purulent collections. Magnetic resonance imaging has been indispensable for the in-depth study of deep tissues. Drainage procedures in both cases allowed for diagnostic sampling of purulent material for a microbiological analysis. Simultaneously, they play a fundamental therapeutic role.

Regarding microbiological investigations, in the first case, direct microscopy revealed Gram-positive cocci, and species identification was performed using MALDI-TOF mass spectrometry. In the second case, an etiological diagnosis was established through a culture examination performed using peripheral blood. Both techniques (MALDI-TOF mass spectrometry and culture examination) are frequently used in clinical practice owing to their high concordance rates [43,44,45].

Despite culturing for both aerobic and anaerobic bacteria from deep tissue specimens, no other pathogens were identified in either case, except for the ubiquitous skin commensal, *Cutibacterium acnes* (isolated from the drainage pus sample) [46].

The microbial isolates were subsequently tested for antimicrobial susceptibility; in both cases, *S. intermedius* demonstrated susceptibility to ampicillin, penicillin, and ceftriaxone, confirming the high penicillin susceptibility rates of SAGs [47]. Indeed, Furuichi and Horikoshi (2018) reported a complete susceptibility of *S. intermedius* to penicillin, ampicillin, cefotaxime, erythromycin, clindamycin, levofloxacin, and vancomycin in pediatric cases [29]. Tetracycline resistance was observed in the bacterial isolate from case number one, which is consistent with the well-documented high rates of tetracycline and, to a lesser extent, macrolide resistance among this group of Streptococci [48,49].

Given the suspicion of bacterial infections based on clinical presentation and in initial investigations, both patients were started on broad-spectrum empiric antibiotics without delay.

In case number one, a third-generation cephalosporin was administered parenterally in combination with metronidazole to broaden the spectrum of activity against anaerobic organisms. Additionally, given the significant size of the abscess (dimensions of 65 × 53 × 74 mm), ultrasound-guided needle aspiration was performed not only for diagnostic purposes but also to evacuate a great portion of the collection.

Regarding case number two, upon admission to our ward, a combined three-antibiotic therapy with ceftriaxone, vancomycin, and metronidazole was initiated parenterally since the polymicrobial spectrum of bacteria is typical for acute mediastinitis [50]. Gram-positive bacteria are responsible for the majority of cases, followed by anaerobic organisms [51].

The 5-week course of antibiotic therapy, which included both intravenous and oral administration, resulted in an excellent clinical response in both cases, as supported by the latest evidence [52,53].

Drainage and physical evacuation of purulent collections played a primary role in the healing process in combination with antibiotics.

Follow-up examinations, including medical evaluations and imaging, revealed a remarkable recovery, with full restoration of anatomical and functional integrity of the previously infected areas. We believe that the absence of comorbidities and the patients’ young ages were crucial factors in achieving a successful and complete recovery in addition to early antibiotic therapy and evacuative treatments.

## 4. Conclusions

*S. intermedius* is no longer considered a mere commensal but rather an opportunistic pathogen. While disseminated infections caused by these organisms are more common in individuals with risk factors (both local and systemic), they can occasionally be observed in otherwise healthy patients, including children. *S. intermedius* demonstrates good susceptibility to beta-lactams both in vitro and in clinical practice. However, great attention must be paid to these invasive and/or disseminated infections as they can still lead to severe and life-threatening conditions.

## Figures and Tables

**Table 1:** Laboratory values of blood tests performed during hospital stay.

	Day 0	Day 3	Day 8	Day 14	Day 17
Leukocyte count (×10^9^/L)	15.280	11.480	12.410	7.27	4.57
Platelets count (×10^9^/L)	134	188	654	757	319
C-reactive protein (mg/L)	236.9	218.4	81.9	10.5	5
Procalcitonin (ng/mL)	10.9	8.3	0.79	-	-
SGPT (IU/L)	120	49	39	29	-
SGOT (IU/L)	81	33	42	33	-
gamma-GT (IU/L)	18	45	70	96	-

Abbreviations: SGPT = serum glutamic pyruvic transaminase; SGOT = serum glutamic oxaloacetic transaminase; gamma-GT = gamma-glutamyl transpeptidase.

## Data Availability

The original contributions presented in this study are included in the article. Further inquiries can be directed to the corresponding author.
